# Chronic troponin elevation assessed by myocardial T1 mapping in patients with stable coronary artery disease

**DOI:** 10.1097/MD.0000000000033548

**Published:** 2023-04-21

**Authors:** Carlos Alexandre W. Segre, James A. de Lemos, Antonildes Nascimento Assunção Junior, Cesar Higa Nomura, Desiderio Favarato, Celia Maria Cassaro Strunz, Alexandre Volney Villa, Jose Rodrigues Parga Filho, Paulo Cury Rezende, Whady Hueb, Jose Antonio Franchini Ramires, Roberto Kalil Filho, Carlos Vicente Serrano Junior

**Affiliations:** a Heart Institute (InCor) University of São Paulo Clinics Hospital, Sao Paulo, Brazil; b Division of Cardiology, Department of Medicine, University of Texas Southwestern Medical Center, Dallas, TX.

**Keywords:** cardiac troponin, cardiovascular magnetic resonance (CMR), chronic coronary artery disease, chronic troponin elevation, stable angina, T1 mapping

## Abstract

Cardiac troponin detected with sensitive assays can be chronically elevated, in the absence of unstable coronary syndromes. In patients with chronic coronary artery disease, clinically silent ischemic episodes may cause chronic troponin release. T1 mapping is a cardiovascular magnetic resonance technique useful in quantitative cardiac tissue characterization. We selected patients with anatomically and functionally normal hearts to investigate associations between chronic troponin release and myocardial tissue characteristics assessed by T1 mapping.

**Methods:**

We investigated the relationship between cardiac troponin I concentrations and cardiovascular magnetic resonance T1 mapping parameters in patients with stable coronary artery disease enrolled in MASS V study before elective revascularization. Participants had no previous myocardial infarction, negative late gadolinium enhancement, normal left ventricular function, chamber dimensions and wall thickness.

**Results:**

A total of 56 patients were analyzed in troponin tertiles: nativeT1 and extracellular volume (ECV) values (expressed as means ± standard deviations) increased across tertiles: nativeT1 (1006 ± 27 ms vs 1016 ± 27 ms vs 1034 ± 37 ms, ptrend = 0.006) and ECV (22 ± 3% vs 23 ± 1.9% vs 25 ± 3%, ptrend = 0.007). Cardiac troponin I concentrations correlated with native T1(*R* = 0.33, *P* = .012) and ECV (*R* = 0.3, *P* = .025), and were independently associated with nativeT1 (*P* = .049) and ventricular mass index (*P* = .041) in multivariable analysis.

**Conclusion:**

In patients with chronic coronary artery disease and structurally normal hearts, troponin I concentrations correlated with T1 mapping parameters, suggesting that diffuse edema or fibrosis scattered in normal myocardium might be associated with chronic troponin release.

## 1. Introduction

In stable patients, detectable troponin concentrations (above the limit of detection and beyond the 99^th^ percentile)^[1]^ have become common with high sensitivity assays^[2]^ and are associated with the presence of coronary artery disease (CAD)^[3]^ and the incidence of cardiovascular death, heart failure,^[4,5]^ and all-cause mortality.^[6]^ Different mechanisms of chronic troponin release have been described in patients with structural heart disease (stretch related injury, altered calcium metabolism)^[7]^ and normal ventricular structure (inflammation and demand ischemia).^[8]^ Specifically in patients with chronic coronary artery disease, clinically silent ischemic episodes, due to small vessel occlusion^[4]^ and plaque microembolization^[9]^ may cause chronic troponin release. If these ischemic episodes would cause edema or focal areas of fibrosis, a very sensitive imaging method like cardiovascular magnetic resonance (CMR) could detect these subtle tissue alterations,^[10]^ and find tissue clues of chronic troponin release.

Late gadolinium enhancement (LGE) by CMR is an accurate method to detect myocardial fibrosis.^[11]^ However, LGE techniques have limitations in patients with diffuse myocardial damage.^[12]^ Innovations in CMR allow the acquisition of quantitative measures of myocardial and blood T1, visualized as a T1 map: native T1 obtained before gadolinium administration can detect and quantify diffuse and focal pathological processes.^[13]^ Contrast T1 composed of images obtained after gadolinium administration can be subtracted from native T1 to assess the myocardial extracellular volume (ECV) that corresponds to gadolinium distribution,^[14]^ enabling the detection of interstitial edema and fibrosis,^[15]^ and infarction areas in patients with chronic coronary artery disease.^[16]^ Therefore, T1 mapping and ECV can detect subtle alterations in otherwise normal hearts, differentiating between cellular and interstitial alterations. by T1 mapping has been shown to be relevant in risk stratification in chronic CAD patients,

Prognostic relevance (above risk scores and left ventricular ejection fraction) of T1 mapping characterization of noninfarcted myocardium (areas without LGE) has also been demonstrated in chronic CAD patients: native T1 was an independent predictor of all-cause mortality and the association of native T1 (noninfarcted) and LGE (extent), in a model that avoided multicollinearity, was an independent predictor of MACCE (cardiac mortality, nonfatal acute coronary syndrome, stroke, and appropriate device discharge).^[17]^ The prognostic relevance of T1 mapping has also been demonstrated, in a 4-years follow-up study after acute ST-segment elevation myocardial infarction: both infarcted and noninfarcted T1 were independent predictors of MACE (cardiac death, sustained ventricular arrhythmia, and new-onset heart failure) and significantly improved risk prediction beyond left ventricular ejection fraction, infarct size, and microvascular obstruction.^[18]^

In summary, in stable patients, chronic troponin elevation is common, and may have prognostic value.^[5,6]^ T1 mapping in infarcted and noninfarcted myocardium areas has also been demonstrated to have prognostic relevance.^[17,18]^ In structurally normal hearts, the mechanism of chronic troponin release and its prognostic relevance may be associated with scattered myocardial tissue alterations that can be assessed by CMR T1 mapping and were not previously appreciated with conventional methods. This possible association has not yet been investigated and may help to understand the anatomical substrate for troponin elevation in chronic patients.

## 2. Methods

### 2.1. Patients

The patients included in this study, were selected from the MASS V trial which compared biomarkers and CMR before and after coronary revascularization procedures. Patients included had multivessel CAD, stable angina symptoms, and normal left ventricular ejection fraction with a formal indication for revascularization.^[19]^

In the present study, we analyzed troponin I concentrations in association with CMR T1 parameters before the proposed intervention. Previous myocardial infarction was not an exclusion criterion in the MASS V study. A scar from a previous myocardial infarction and detectable LGE can alter troponin concentrations and ECV,^[20]^ therefore, we excluded all patients with detectable LGE by CMR in this analysis (n = 42). Patients with hypertrophic, infiltrative, Chagas disease or any other kind of cardiomyopathy were also excluded.

The study conformed to the guidelines set out in the Declaration of Helsinki, and all participants gave written informed consent. The Institutional Ethics Committee of the University of São Paulo approved the study.

### 2.2. Laboratory analysis

Blood samples were collected from each patient before the revascularization procedure was undertaken. Glucose, total cholesterol, HDL-cholesterol, LDL cholesterol, triglycerides, High Sensitivity C-reactive protein, and CKMB were determined with standard procedures.

Cardiac troponin I was determined using the ADVIA Centaur TnI-Ultra kit (Siemens Healthcare Diagnostics, NY) in the automated equipment of the same manufacturer. The test is an immunoassay that uses a direct chemiluminescence technology and constant amounts of 2 monoclonal antibodies. An increased troponin I concentration was defined as a value exceeding the limit of detection of the method and below the 99^th^ of a reference population. According to the manufacturers, the detection limit is 6 ng/L, and the population reference value at the 99^th^ percentile is 40 ng/L, the coefficient of variation is < 10% at the 99^th^ percentile. Siemens Advia Centaur ultra-troponin I kit is designated as a contemporary or level 1 troponin assay, corresponding to <50% of normal individuals with detectable troponin concentrations.^[21]^

### 2.3. Cardiovascular magnetic resonance protocol

CMR examinations were performed using a clinical 1.5T scanner (Philips Achieva, Best, The Netherlands). Standard cine-imaging with steady state free precession pulse was used to acquire left ventricle (LV) short-axis images for quantification of ventricular volumes, mass and function. Late gadolinium enhancement was also performed using standard segmented IR prepared gradient-echo sequence 10 to 15 minutes after an intravenous bolus injection (0.1 mmol/kg) of Gadoterate meglumine Gd-DOTA (Guerbet SA, France). T1 mapping was performed using an ECG-triggered single-shot modified look-locker inversion recovery sequence with a 3 (3) 3 (3) 5 sampling pattern and the following parameters: slice thickness 10 mm, field of view 300 × 300 mm, ACQ matrix (read-out × phase-encodings) 152 × 150, flip angle 40, minimum TI 60 ms, inversion-time increment 150 ms. A mid modified look-locker inversion recovery short-axis image was acquired prior, and 15 minutes after the intravenous bolus of contrast.

### 2.4. Image post-processing

**Figure 1.**
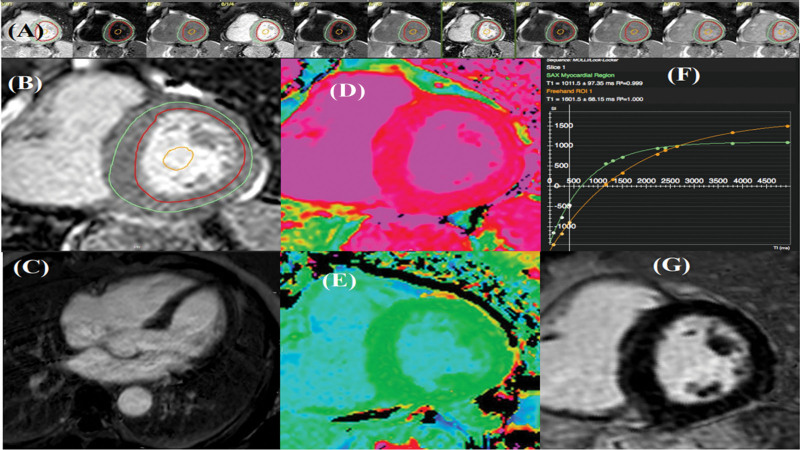
T1 mapping - mid-ventricular wall. (A) MOLLI sequence acquisition. (B) LV planimetry. (C) Normal 4 chamber view LGE image. (D, E) T1 mapping. (F, G) T1 mapping Graph Normal SA LGE image. LGE = late gadolinium enhancement, LV = left ventricle, MOLLI = modified look-locker inversion recovery.

CMR images were analyzed using the CVI42 software (Circle Cardiovascular Imaging Inc. Calgary, Canada) by experienced imaging experts (AVV, AAJ, JP) who were unaware of clinical or laboratory data. End systolic, end-diastolic LV volumes, LV mass and LV ejection fraction were calculated using standard methods. For T1, a region of interest was manually drawn at mid-ventricular level, sufficiently far from tissue interfaces, for both myocardial and LV cavity blood, as depicted in Figure 1. The Extracellular volume was calculated using native and contrast T1 values, and hematocrit, which was drawn simultaneously with the CMR study.

### 2.5. Statistical analysis

Variables are expressed as means ± SD and frequency (percentage). Normality assumption was assessed graphically using QQ plot. Patients were split in tertiles of troponin I concentrations. Differences across tertiles were assessed using 1-way analysis of variance (ANOVA) for normally distributed variables, Kruskal-Wallis for variables with non-normal distribution and chi-square for proportions.

Correlations between troponin I concentrations and T1 mapping parameters were determined using Spearman correlation coefficient. A multivariable linear regression analysis was performed to investigate the association between troponin concentrations and native T1/ECV adjusted for age and sex,^[22]^ as well as for the significant variables found in the univariate model. Since native T1 and ECV are correlated (multicollinearity), results for both variables were analyzed in 2 different multivariable models. All statistical analyses were performed using R version 3.5.3 (R Foundation for Statistical Computing, Vienna, Austria) and a *P* value < .05 was considered statistically significant.

## 3. Results

From a total of 202 patients who underwent CMR, T1 mapping was performed in 98 patients and 56 (mean age 63 ± 8 years, 68% male) with negative LGE were selected for the current study. A total of 37 (66%) of the patients had detectable troponin concentrations (above 0.006 mg/dL).

**Figure 2.**
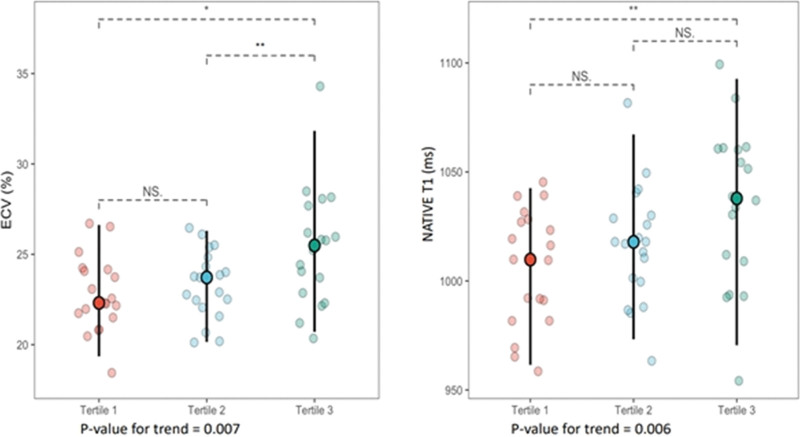
ECV and NativeT1 across troponin tertiles (*P < .05, **P < .01). ECV = extracellular volume.

Baseline characteristics across troponin tertiles are shown in Table 1. Diabetes mellitus prevalence was higher in tertiles 2 and 3 compared to tertile 1 (*P* = .003). LV volumes and function were similar across tertiles. LV mass increased across tertiles with a marginally significant *P* = .062. ECV and native T1 (expressed as means ± standard deviations) increased in a stepwise fashion across cardiac troponin I (cTnI) tertiles: ECV (22 ± 3% vs 23 ± 1.9% vs 25 ± 3%, ptrend = 0.007) and nativeT1 (1006 ± 27ms vs 1016 ± 27 ms vs 1034 ± 37 ms, ptrend = 0.006), as illustrated in Figure 2. Cardiac dimensions and function determined by CMR were within the normal range of the method used.^[23]^

**Table 1:** Patient’s baseline characteristics across troponin tertiles.

All patients		Tertile 1	Tertile 2	Tertile 3	*P* value
(n = 56)		(n = 19)	(n = 19)	(n = 18)	
Clinical					
Age, yr	64 ± 8	64 ± 8	62 ± 7	65 ± 9	.513
Male, n (%)	38 (68)	13 (69)	12 (58)	14 (78)	.629
BMI, kg/m2	28 ± 5	30 ± 4	27 ± 4	28 ± 6	.148
Diabetes mellitus, n (%)	30 (54)	3 (27.3)	15 (62.5)	11 (52.4)	.003
Hypertension, n (%)	31 (55)	9 (47)	12 (63)	10 (56)	.619
Current - previous smoking, n (%)	32 (57)	13 (68)	10 (53)	12 (67)	.546
Laboratory					
CRP, mg/L (M, IQ)	3.77 (1.80–7.04)	4.27 (2.29–7.09)	2.04 (1.41–5.32)	5.58 (2.58–13.54)	>.05
CK, U/L (M, IQ)	94,5 (74.5–176.3)	95 (76.5–136.5)	95 (75–152.5)	91.5 (67.5–119.3)	>.05
CKMB, U/L (M, IQ)	1.27 (0.72–1.73)	1.41 (0.69–1.80)	1.24 (0.9–1.66)	1.27 (0.74–1.79)	>.05
Hematocrit, % (M, IQ)	43 (40–46,25)	45.0 (40.5–47)	42.0 (40.0–45.5)	42.5 (41.0–45.0)	>.05
Cr, mg/dL (M, IQ)	1.0 (0.89–1.10)	0.97 (0.91–1.07)	1.00 (0.83–1.08)	1.03 (0.91–1,18)	>.05
CMR					
Septum, mm	9.5 ± 1.7	9.1 ± 1.2	9.6 ± 1.8	9.5 ± 1.8	.607
LA, mm	34 ± 5	33 ± 5	35 ± 4	36 ± 6	.191
LVEDV index, ml/m2	67 ± 20	65 ± 21	63 ± 12	72 ± 24	.357
LVESV index, ml/m2	23 ± 11	23 ± 13	21 ± 6	25 ± 12	.537
LVEF, %	66 ± 8	67 ± 9	67 ± 6	65 ± 9	.691
LV mass index, ml/m2	63 ± 16	59 ± 10	61 ± 17	70 ± 16	.062
Native T1, ms	1019 ± 32	1006 ± 27	1016 ± 27	1034 ± 37	.025
Contrast T1, ms	479 ± 47	472 ± 41	475 ± 44	492 ± 57	.397
ECV, %	24 ± 3	22 ± 2	23 ± 1.9	25 ± 3	.001

BMI = body mass index, CCS = Canadian Cardiovascular Society, CMR = cardiovascular magnetic resonance, ECV = extracellular volume fraction, eGFR = estimated glomerular filtration rate, LA = left atrium, LV = left ventricle, LVEDV = Left ventricular end–diastolic volume, LVESV = Left ventricular end systolic volume.

**Figure 3.**
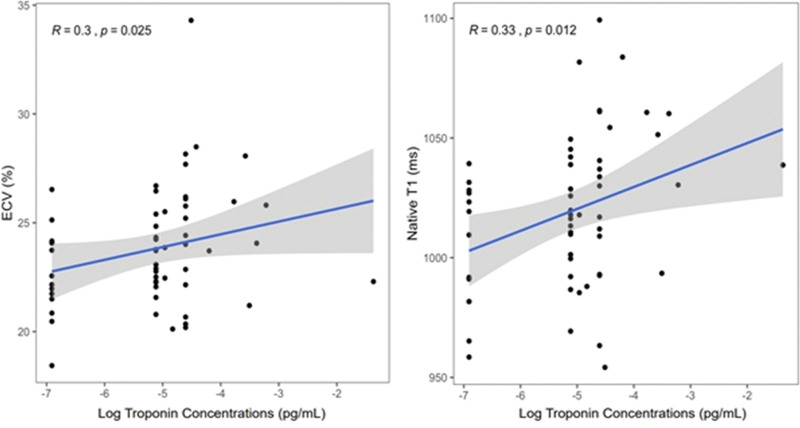
Correlations between troponin concentrations and T1 mapping parameters.

Figure 3 displays the correlations between troponin concentrations and ECV (*R* = 0.30, *P* = .025) and native T1 (*R* = 0.33, *P* = .012). As depicted in Table 2, in univariable linear regression analysis, cTnI concentrations, assessed as a continuous variable, were significantly associated with ECV (*P* = .049), native T1 (*P* = .012), and LV mass index (*P* = .014). In multivariable linear regression analysis troponin concentrations were independently associated with LV mass index (*P* = .041) native T1 (model 1, *P* = .049) but not with ECV (model 2).

**Table 2:** Univariable and multivariable linear regression analysis for troponin concentrations.

	Univariable	Model 1	Model 2
	Estimate (95% CI)	Estimate (95% CI)	Estimate (95% CI)
	*P* value	*P* value	*P* value
Age†	0.15 (−0.22 to 0.53)	0.12 (−0.24 to 0.49)	0.14 (−0.22 to 0.51)
	*P* = .429	*P* = .520	*P* = .447
Female	−0.26 (−0.92 to 0.38)	−0.26 (−0.92 to 0.39)	−0.09 (−0.80 to 0.63)
	*P* = .426	*P* = .434	*P* = .822
Diabetes	0.30 (−0.31 to 0.91)		
	*P* = .335		
Hypertension	0.21 (−0.40 to 0.83)		
	*P* = .496		
Smoking	0.31 (−0.32 to 0.93)		
	*P* = .343		
BMI*	−0.17 (−0.49 to 0.15)		
	*P* = .293		
LVEF*	0.02 (−0.17 to −0.21)		
	*P* = .865		
LV mass index‡	0.24 (0.05 to 0.43)	0.21 (0.01 to 0.41)	0.17 (−0.05 to 0.39)
	*P* = .014	*P* = .041	*P* = .139
Native T1‡	0.60 (0.15 to 1.06)	0.49 (0.01 to 0.99)	
	*P* = .012	*P* = .049	
ECV*	0.55 (0 to 1.11)		0.36 (−0.25 to 0.97)
	*P* = .049		*P* = .253

ECV = extracellular volume, LV = left ventricle.

* 5 units increase.

† 10 units increase.

‡ 50 units increase.

**Figure 4.**
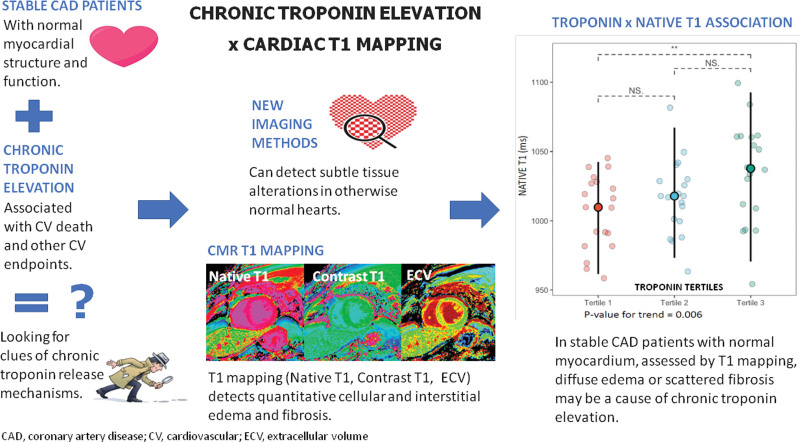
Chronic troponin elevation assessed by myocardial T1 mapping in coronary artery disease.

The main idea of this study and its results are shown in Figure 4.

## 4. Discussion

In the present study, we evaluated 56 patients with stable, multivessel CAD, normal ventricular chamber dimensions, normal wall thickness, and negative LGE by CMR. The specific contribution of this study is to show that among patients with structurally normal hearts, differences detected by T1 mapping CMR were still found across troponin tertiles as reflected by higher ECV and native T1 and an independent association with LV mass index and native T1. Therefore, focal areas of edema or fibrosis in myocardial cells or interstitium might be associated with troponin elevation.

Previous studies that investigated T1 mapping and troponin analyzed patients with myocardial scars.^[24,25]^ In this study, we selected stable patients without detectable LGE and normal cardiac chambers size and function, to investigate troponin release in the absence of myocardial scars. In this setting, this is the first study to show an association between chronic troponin release, ECV, and native T1 by CMR.

Consistent with our findings, other studies have also found an association between myocardial damage detected with different CMR techniques and high sensitivity troponin elevation. In patients with hypertrophic cardiomyopathy analyzed with CMR T2-weighted imaging there was correlation between myocardial injury and high sensitivity troponin T levels.^[26]^ In patients initially free of cardiovascular disease who underwent serial cardiovascular magnetic resonance with late gadolinium enhancement, there was an association of high sensitivity troponin T levels with progressive fibrosis.^[27]^ It is possible that the current study findings with T1 mapping detect the very initial phase of this fibrosis process. The mechanism of diffuse myocardial damage might also explain the independent association of high sensitivity troponin I levels and incident heart failure in the general population.^[28]^ Diffuse myocardial injury detected by T1 mapping is associated with subtle ventricular dysfunction in diabetic patients^[29]^ and diastolic dysfunction in those with hypertrophic cardiomyopathy,^[30]^ associations that might also help to understand the worse clinical prognosis of stable CAD patients even with minor troponin I releases.^[31]^ As a future perspective, studies in chronic patients that associate high sensitivity troponin, T1 and T2 mapping may improve risk stratification and help to predict prognosis in the long-term.

Diabetes mellitus prevalence was higher in troponin tertiles 2 and 3 compared to tertile 1. This finding is consistent with the importance of diabetes mellitus as a predictor of chronic troponin elevation in the general population^[32]^ but also defines a subset of patients at a higher risk of cardiovascular events: chronic troponin elevation in diabetic patients is associated with the presence of CAD,^[3]^ with an increased risk of cardiovascular death, myocardial infarction and cardiovascular death,^[33]^ and with all-cause death, myocardial infarction and stroke in patients with limb ischemia with and without CAD.^[34]^ In this higher risk group of patients, there is an increase in native T1 and ECV which can be caused by the summed damage of CAD, in epicardial coronary arteries and microvascular disease associated with diabetes mellitus.^[35]^ A similar association was shown in another study in which a subset of patients with preserved ventricular function increased ECV and high troponin and brain natriuretic peptide concentrations were at higher risk of incident heart failure hospitalization, and death from any cause.^[25]^

Although ECV had a consistent correlation with troponin increase, it was not associated with troponin concentrations in multivariable analysis. The probable reason for this lack of association is the relatively small number of patients. However, it is also possible that chronic troponin elevation is more strongly associated with changes at the cellular level in cardiomyocytes, as reflected by increasing native T1. The lack of a T2 mapping sequence is a limitation of this study to a better interpretation of ECV.

There was also a consistent association of troponin elevation with left ventricular mass. As 55% of the patients were hypertensive and ventricular hypertrophy is associated with troponin elevation,^[36]^ this is not an unexpected finding. However, it is important to note that ventricular mass measurements were within the normal range, which indicates that troponin is a biomarker of subtle alterations in myocardial structure.

Limitations to this study are the small number of patients evaluated, and the relatively small number of patients with T1 mapping in the MASS V study. T1 mapping was not a predetermined measurement in the MASS V study, which used cardiovascular magnetic resonance with late gadolinium enhancement to detect myocardial infarction. Previous myocardial infarction was neither an exclusion criterion if it did not compromise ventricular function. These factors may have led to a relatively small final number of patients with T1 mapping and LGE negative CMR.

Another possible criticism is that ECV values determined in our stable CAD patients were similar to those found in normal individuals in other studies. Reported values of ECV in normal myocardium are in the range of 24% to 28%,^[37]^ but this range may be broader, between 22% and 32%,^[38]^ or narrower, between 21.7% and 26.2%.^[39]^ These values may vary according to the equipment and specific technique used.^[40]^ The lack of a control group to determine normal ECV values with the method used is a limitation of this study. Despite, there was a stepwise increase in ECV values across troponin tertiles, and the modest differences are consistent with differences found in another study.^[40]^ It is also important to notice that to quantify T1 values with current techniques, a region of interest is drawn in a ventricular slice. This specific region is a sample, and its alterations may not correspond to the whole left ventricle characteristics,^[41]^ nevertheless, MASS V^[19]^ study included patients with multivessel disease who have diffuse ischemia and probably diffuse tissue alterations.

Finally, because we have only a single troponin I measurement performed before revascularization, we cannot be certain that some individuals did not have acute, rather than chronic cTnI elevation, nevertheless none of the patients had symptoms of recent onset, rapidly progressive or unstable angina.

This is a cross-sectional study that has generated a hypothesis about scattered areas of myocardial injury and troponin release in stable CAD patients. As is well known, an inherent limitation of this type of study is that it cannot provide definitive information about cause-and-effect relationships. Additional studies with a larger sample of patients are required to confirm these findings.

## 5. Conclusion

In this sample of patients, we found an independent association between troponin concentrations, native T1 and ventricular mass index and a correlation with CMR T1 mapping parameters, native T1 and ECV. These new findings indicate that focal areas of edema or fibrosis scattered in normal myocardium might be the anatomical substrate for chronic troponin release in stable coronary artery disease patients.

## Author contributions

**Conceptualization:** Carlos A W Segre, Célia Maria Cassaro Strunz, Whady Hueb.

**Data curation:** Antonildes N Assunção Junior, Cesar Higa Nomura, Desiderio Favarato, Alexandre V Villa, José Rodrigues Parga Filho.

**Formal analysis:** Carlos A W Segre, Antonildes N Assunção Junior, Cesar Higa Nomura, Desiderio Favarato, Célia Maria Cassaro Strunz, Alexandre V Villa, José Rodrigues Parga Filho.

**Funding acquisition:** Whady Hueb.

**Methodology:** Carlos A W Segre, Célia Maria Cassaro Strunz.

**Supervision:** James A De Lemos, Whady Hueb.

**Writing – original draft:** Carlos A W Segre.

**Writing – review & editing:** Carlos A W Segre, James A De Lemos, Desiderio Favarato, Paulo Cury Rezende, Whady Hueb, Jose A F Ramires, Roberto Kalil Filho, Carlos Vicente Serrano Junior.

## References

[1] Aw TC Huang WT Le TT. High-sensitivitycardiac troponinsin cardio-healthy subjects: a cardiovascular magnetic resonance imaging study. Sci Rep. 2018;8:15409. 30337631 10.1038/s41598-018-33850-9 PMC6194119

[2] Apple FS Ler R Murakami MM. Determination of 19 cardiac troponin I and T assay 99th percentile values from a common presumably healthy population. Clin Chem. 2012;58:1574–81. 22983113 10.1373/clinchem.2012.192716

[3] Segre CA Hueb W Garcia RM. Troponin in diabetic patients with and without chronic coronary artery disease. BMC Cardiovasc Disord. 2015;15:72. 26195004 10.1186/s12872-015-0051-z PMC4508806

[4] Omland T de Lemos JA Sabatine MS. A sensitive cardiac troponin T assay in stable coronary artery disease. N Engl J Med. 2009;361:2538–47. 19940289 10.1056/NEJMoa0805299 PMC2997684

[5] Omland T Pfeffer MA Solomon SD. Prognostic value of cardiac troponin I measured with a highly sensitive assay in patients with stable coronary artery disease. J Am Coll Cardiol. 2013;61:1240–9. 23414791 10.1016/j.jacc.2012.12.026

[6] Giannitsis E Spanuth E Horsch A. High-sensitivity cardiac troponin T and N-terminal pro-B-type natriuretic peptide predict mortality in stable coronary artery disease: results from the Ludwigshafen Risk and Cardiovascular Health (LURIC) study. Clin Chem Lab Med. 2013;51:2019–28. 24072576 10.1515/cclm-2012-0786

[7] Kociol RD Pang PS Gheorghiade M. Troponin elevation in heart failure prevalence, mechanisms, and clinical implications. J Am Coll Cardiol. 2010;56:1071–8. 20863950 10.1016/j.jacc.2010.06.016

[8] Frencken JF van Baal L Kappen TH. Myocardial injury in critically ill patients with community-acquired pneumonia. a cohort study. Ann Am Thorac Soc. 2019;16:606–12. 30521759 10.1513/AnnalsATS.201804-286OC

[9] Korosoglou G Lehrke S Mueller D. Determinants of troponin release in patients with stable coronary artery disease: insights from CT angiography characteristics of atherosclerotic plaque. Heart. 2011;97:823–31. 20884786 10.1136/hrt.2010.193201

[10] Adam RD Shambrook J Flett AS. The prognostic role of tissue characterisation using cardiovascular magnetic resonance in heart failure. Card Fail Rev. 2017;3:86–96. 29387459 10.15420/cfr.2017:19:1 PMC5739890

[11] Mewton N Liu CY Croisille P. Assessment of myocardial fibrosis with cardiovascular magnetic resonance. J Am Coll Cardiol. 2011;57:891–903. 21329834 10.1016/j.jacc.2010.11.013 PMC3081658

[12] Fernandes JL Rochitte CE. T1 mapping: technique and applications. Magn Reson Imaging Clin N Am. 2015;23:25–34. 25476671 10.1016/j.mric.2014.08.007

[13] Messroghli DR Moon JC Ferreira VM. Clinical recommendations for cardiovascular magnetic resonance mapping of T1, T2, T2* and extracellular volume: a consensus statement by the society for cardiovascular magnetic resonance (SCMR) endorsed by the European association for cardiovascular imaging (EACVI). J Cardiovasc Magn Reson. 2017;19:75. 28992817 10.1186/s12968-017-0389-8 PMC5633041

[14] Puntmann VO Peker E Chandrashekhar Y. T1 mapping in characterizing myocardial disease: a comprehensive review. Circ Res. 2016;119:277–99. 27390332 10.1161/CIRCRESAHA.116.307974

[15] Haaf P Garg P Messroghli DR. Cardiac T1 mapping and extracellular volume (ECV) in clinical practice: a comprehensive review. J Cardiovasc Magn Reson. 2016;18:89. 27899132 10.1186/s12968-016-0308-4 PMC5129251

[16] Ugander M Oki AJ Hsu LY. Extracellular volume imaging by magnetic resonance imaging provides insights into overt and sub-clinical myocardial pathology. Eur Heart J. 2012;33:1268–78. 22279111 10.1093/eurheartj/ehr481 PMC3350985

[17] Puntmann VO Carr-White G Jabbour A. Native T1 and ECV of noninfarcted myocardium and outcome in patients with coronary artery disease. J Am Coll Cardiol. 2018;71:766–78. 29447739 10.1016/j.jacc.2017.12.020

[18] Shanmuganathan M Masi A Burrage MK. Acute response in the noninfarcted myocardium predicts long-term major adverse cardiac events after STEMI. JACC Cardiovasc Imag. 2023;16:46–59. 10.1016/j.jcmg.2022.09.015 PMC9834063 36599569

[19] Hueb W Gersh BJ Rezende PC. Hypotheses, rationale, design, and methods for prognostic evaluation of cardiac biomarker elevation after percutaneous and surgical revascularization in the absence of manifest myocardial infarction. A comparative analysis of biomarkers and cardiac magnetic resonance. The MASS-V Trial. BMC Cardiovasc Disord. 2012;12:65. 22898311 10.1186/1471-2261-12-65 PMC3468382

[20] Wong TC Piehler KM Kang IA. Myocardial extracellular volume fraction quantified by cardiovascular magnetic resonance is increased in diabetes and associated with mortality and incident heart failure admission. Eur Heart J. 2014;35:657–64. 23756336 10.1093/eurheartj/eht193 PMC3945798

[21] Apple FS. A new season for cardiac troponin assays: it’s time to keep a scorecard. Clin Chem. 2009;55:1303–6. 19478023 10.1373/clinchem.2009.128363

[22] Neilan TG Coelho-Filho OR Shah RV. Myocardial extracellular volume fraction from T1 measurements in healthy volunteers and mice: relationship to aging and cardiac dimensions. JACC Cardiovasc Imag. 2013;6:672–83. 10.1016/j.jcmg.2012.09.020 PMC3683385 23643283

[23] Kawel-Boehm N Maceira A Valsangiacomo-Buechel ER. Normal values for cardiovascular magnetic resonance in adults and children. J Cardiovasc Magn Reson. 2015;17:29. 25928314 10.1186/s12968-015-0111-7 PMC4403942

[24] Messroghli DR Walters K Plein S. Myocardial T1 mapping: application to patients with acute and chronic myocardial infarction. Magn Reson Med. 2007;58:34–40. 17659622 10.1002/mrm.21272

[25] Yang EY Khan MA Graviss EA. Relationship of extracellular volume assessed on cardiac magnetic resonance and serum cardiac troponins and natriuretic peptides with heart failure outcomes. Sci Rep. 2019;9:20168. 31882822 10.1038/s41598-019-56213-4 PMC6934524

[26] Chen S Huang L Zhang Q. T2-weighted cardiac magnetic resonance image and myocardial biomarker in hypertrophic cardiomyopathy. Medicine (Baltim). 2020;99:e20134e20134. 10.1097/MD.0000000000020134 PMC7306317 32501969

[27] Seliger SL Hong SN Christenson RH. High-sensitive cardiac troponin t as an early biochemical signature for clinical and subclinical heart failure: MESA (multi-ethnic study of atherosclerosis). Circulation. 2017;135:1494–505. 28159799 10.1161/CIRCULATIONAHA.116.025505 PMC5401621

[28] Yan I Borschel CS Neumann JT. High-sensitivity cardiac troponin i levels and prediction of heart failure: results from the BiomarCaRE consortium. JACC Heart Fail. 2020;8:401–11. 32171759 10.1016/j.jchf.2019.12.008

[29] Ellims AH Iles LM Ling LH. Diffuse myocardial fibrosis in hypertrophic cardiomyopathy can be identified by cardiovascular magnetic resonance, and is associated with left ventricular diastolic dysfunction. J Cardiovascular Magnetic Resonance. 2012;14:76. 10.1186/1532-429X-14-76 PMC3502601 23107451

[30] Ng AC Auger D Delgado V. Association between diffuse myocardial fibrosis by cardiac magnetic resonance contrast-enhanced T (1) mapping and subclinical myocardial dysfunction in diabetic patients: a pilot study. Circ Cardiovasc Imag. 2012;5:51–9. 10.1161/CIRCIMAGING.111.965608 22135399

[31] Melki D Lugnegard J Alfredsson J. Implications of introducing high-sensitivity cardiac troponin T into clinical practice: data from the SWEDEHEART registry. J Am Coll Cardiol. 2015;65:1655–64. 25908071 10.1016/j.jacc.2015.02.044

[32] Hallen J Johansen OE Birkeland KI. Determinants and prognostic implications of cardiac troponin T measured by a sensitive assay in type 2 diabetes mellitus. Cardiovasc Diabetol. 2010;9:52. 20843304 10.1186/1475-2840-9-52 PMC2946276

[33] Everett BM Cook NR Magnone MC. Sensitive cardiac troponin T assay and the risk of incident cardiovascular disease in women with and without diabetes mellitus: the women’s health study. Circulation. 2011;123:2811–8. 21632491 10.1161/CIRCULATIONAHA.110.009928 PMC3144564

[34] Cimaglia P Dalla Paola L Carone A. High-sensitivity cardiac troponin predicts major cardiovascular events in diabetic patients with critical limb ischemia and foot lesions. Front Cardiovasc Med. 2021;8:595701. 34124184 10.3389/fcvm.2021.595701 PMC8192711

[35] Laakso M. Heart in diabetes: a microvascular disease. Diabetes Care. 2011;34 Suppl 2(Suppl 2):S145–149. 21525446 10.2337/dc11-s209 PMC3632152

[36] Siciliano M Mettimano M Dondolini-Poli A. Troponin I serum concentration: a new marker of left ventricular hypertrophy in patients with essential hypertension. Ital Heart J. 2000;1:532–5. 10994933

[37] Salerno M Kramer CM. Advances in parametric mapping with CMR imaging. JACC. Cardiovascular Imag. 2013;6:806–22. 10.1016/j.jcmg.2013.05.005 PMC4073213 23845576

[38] Liu CY Liu YC Wu C. Evaluation of age-related interstitial myocardial fibrosis with cardiac magnetic resonance contrast-enhanced T1 mapping: MESA (multi-ethnic study of atherosclerosis). J Am Coll Cardiol. 2013;62:1280–7. 23871886 10.1016/j.jacc.2013.05.078 PMC3807823

[39] Wong TC Piehler K Meier CG. Association between extracellular matrix expansion quantified by cardiovascular magnetic resonance and short-term mortality. Circulation. 2012;126:1206–16. 22851543 10.1161/CIRCULATIONAHA.111.089409 PMC3464491

[40] Fontana M White SK Banypersad SM. Comparison of T1 mapping techniques for ECV quantification. Histological validation and reproducibility of ShMOLLI versus multibreath-hold T1 quantification equilibrium contrast CMR. J Cardiovasc Magn Reson. 2012;14:88. 23272651 10.1186/1532-429X-14-88 PMC3552758

[41] Perea RJ Ortiz-Perez JT Sole M. T1 mapping: characterisation of myocardial interstitial space. Insights Imag. 2015;6:189–202. 10.1007/s13244-014-0366-9 PMC4376813 25424598

